# Noncoding RNAs as Key Regulators for Cardiac Development and Cardiovascular Diseases

**DOI:** 10.3390/jcdd10040166

**Published:** 2023-04-12

**Authors:** Satoshi Kawaguchi, Bruno Moukette, Taiki Hayasaka, Angela K. Haskell, Jessica Mah, Marisa N. Sepúlveda, Yaoliang Tang, Il-man Kim

**Affiliations:** 1 Department of Anatomy, Cell Biology, and Physiology, Indiana University School of Medicine, Indianapolis, IN 46202, USA; s-kawa@asahikawa-med.ac.jp (S.K.); bruno.moukette@pfizer.com (B.M.); thayasa@iu.edu (T.H.); angehask@indiana.edu (A.K.H.); jmah@iu.edu (J.M.); masepulv@iu.edu (M.N.S.); 2 Vascular Biology Center, Medical College of Georgia, Augusta University, Augusta, GA 30912, USA; yaotang@augusta.edu

**Keywords:** atherosclerosis, cardiac arrhythmia, cardiac development, cardiac fibrosis, cardiac hypertrophy, cardiovascular diseases, myocardial infarction, noncoding RNAs, pulmonary hypertension

## Abstract

Noncoding RNAs (ncRNAs) play fundamental roles in cardiac development and cardiovascular diseases (CVDs), which are a major cause of morbidity and mortality. With advances in RNA sequencing technology, the focus of recent research has transitioned from studies of specific candidates to whole transcriptome analyses. Thanks to these types of studies, new ncRNAs have been identified for their implication in cardiac development and CVDs. In this review, we briefly describe the classification of ncRNAs into microRNAs, long ncRNAs, and circular RNAs. We then discuss their critical roles in cardiac development and CVDs by citing the most up-to-date research articles. More specifically, we summarize the roles of ncRNAs in the formation of the heart tube and cardiac morphogenesis, cardiac mesoderm specification, and embryonic cardiomyocytes and cardiac progenitor cells. We also highlight ncRNAs that have recently emerged as key regulators in CVDs by focusing on six of them. We believe that this review concisely addresses perhaps not all but certainly the major aspects of current progress in ncRNA research in cardiac development and CVDs. Thus, this review would be beneficial for readers to obtain a recent picture of key ncRNAs and their mechanisms of action in cardiac development and CVDs.

## 1. Introduction

Cardiovascular diseases (CVDs), which continue to be one of the main causes of morbidity and mortality in the adult population worldwide, carry a serious economic burden and pose a public health problem [1]. Although considerable progress has been made and a molecular diagnosis of CVDs using panels of the most prevalent genes is in the works, the molecular mechanisms underlying CVDs remain elusive. Noncoding RNAs (ncRNAs) function as regulators of epigenetics or modulate gene expression at the transcriptional or posttranscriptional level. NcRNAs have thus provided an important new perspective on gene regulation in cardiac development and CVDs [2,3].

The best characterized ncRNAs in the heart are microRNAs (miRNAs or miRs) and long ncRNAs (lncRNA). MiRs contribute to the regulation of cardiac genes after transcription by repressing protein-coding genes, whereas lncRNAs elicit their functions by either activating or inhibiting target genes via multiple mechanisms of action. LncRNAs can interact with DNAs, RNAs, or proteins, influencing transcription and posttranscriptional or epigenetic gene regulation [2,3,4,5,6]. Some miRs and lncRNAs have been reported to exhibit developmental stage- or tissue-specific expression patterns [7,8,9], thus potentiating their roles in cardiac development and CVDs.

Because the number of ncRNAs associated with cardiac development and CVDs has increased, more opportunities have arisen to understand ncRNA-mediated regulatory mechanisms by systematic analyses of their targets, functional roles, and associated diseases [10,11]. Regulatory ncRNAs have gained growing interest in the cardiovascular community because of their crucial roles in cardiac development and CVDs. For example, we previously summarized the roles of miRs, lncRNAs, and circular noncoding RNAs (circRNAs) in human diseases [12,13,14,15,16]. Here, we review the most up-to-date literature to highlight ncRNAs that have recently emerged as important regulators in cardiac development and CVDs. After briefly describing the specific features of miRNAs, lncRNAs, and circRNAs, we then discuss current knowledge of the implications of ncRNAs in cardiac development and multiple CVDs, including atherosclerosis, cardiac arrhythmia, cardiac fibrosis, cardiac hypertrophy, myocardial infarction, and pulmonary hypertension. Last, we emphasize the therapeutic potential of ncRNAs and suggest directions for future work.

## 2. Noncoding RNAs

NcRNAs are classified into two groups based on nucleotide length: small ncRNAs (sncRNAs) and lncRNAs. Most sncRNAs consist of miRs with an average of 22 nucleotides, whereas lncRNAs have more than 200 nucleotides and include linear lncRNAs and circRNAs.

### 2.1. MiRNAs

**Figure 1:**
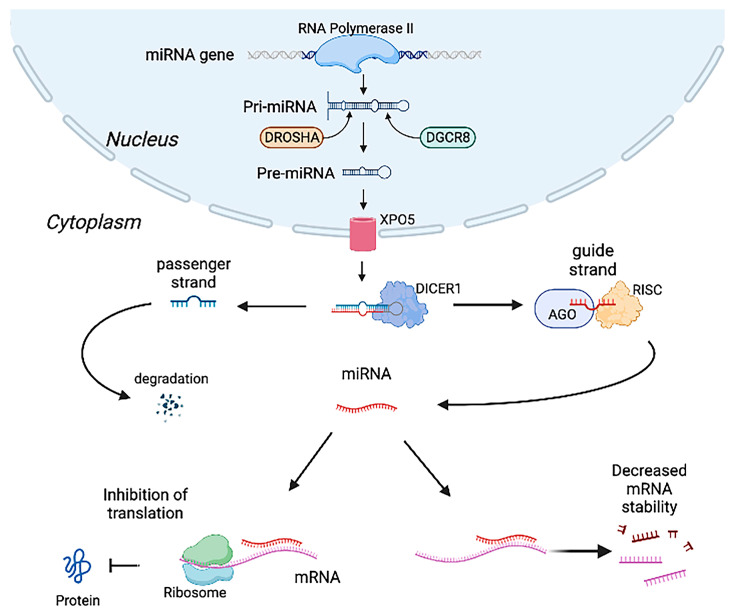
MicroRNA (miRNA) biogenesis and actions. In the nucleus, miRNA is transcribed into the hairpin primary miRNA molecule (pri-miRNA) by RNA polymerase II. Pri-miRNA is processed into the precursor miRNA molecule (pre-miRNA) by DROSHA ribonuclease III (DROSHA) and DiGeorge syndrome chromosomal 8 (DGCR8). Pre-miRNA is transported to the cytoplasm via nucleocytoplasmic protein exportin-5 (XPO5). Pre-miRNA is then cut into 2 of 21–22 nucleotides by an endoribonuclease DICER (DICER1). Although one of the nucleotides (passenger strand) usually undergoes degradation, the other one (guide strand) is incorporated with argonaute (AGO) and RNA-induced silencing complex (RISC) and becomes a mature miRNA. A mature miRNA attenuates protein translation or decreases mRNA stability after binding to target mRNAs.

MiRNAs are a large class of short RNA molecules with 21 to 22 nucleotides. In mammals, miRNAs control about 50% of all protein-coding genes and affect various physiological and pathological processes by modulating cellular pathways [17]. MiRNA biogenesis starts with transcription of hairpin-containing primary miRNA molecules (pri-miRNAs) by RNA polymerase II in the nucleus. The pri-miRNAs are transformed into precursor miRNA molecules (pre-miRNAs) by Drosha ribonuclease III (Drosha) and DiGeorge syndrome chromosomal region 8 (DGCR8). Then, the pre-miRNAs are transported to the cytoplasm through the nucleocytoplasmic protein exportin-5 (XPO5). In the cytoplasm, the pre-miRNAs are cleaved into two strands of 21–22 nucleotides by an endoribonuclease DICER (DICER1). One strand, called the guide strand, is then incorporated with a part of the RNA-induced silencing complex (RISC), interacts with argonaute (AGO), and becomes a mature miRNA. The other, called the passenger strand, usually undergoes accelerated degradation. Some mature miRNAs interact with 5′-untranslated regions (UTRs), coding regions, or gene promoters, but most of them bind to 3′-UTRs of target mRNAs to lead to translational inhibition and/or decreased mRNA stability [18,19,20]. The schematic representation of miRNA biogenesis is shown in Figure 1.

### 2.2. LncRNAs

LncRNAs, which consist of more than 200 nucleotides, are transcribed from intergenic, exonic, or distal protein-coding regions of the genome by RNA polymerase, followed by 3′-polyadenylation and 5′-end capping [21,22]. LncRNAs are functional molecules that play various roles through interaction with mRNAs, miRNAs, DNAs, proteins, and small molecules.

Some lncRNAs in the nucleus regulate downstream genes through chromatin remodeling and histone modifications. Chromatin remodeling is regulated by the SWItch/Sucrose Non-Fermentable (SWI/SNF) complex, which modulates nucleosome localization. Histones also play important roles in chromosome formation and function through methylation and acetylation. LncRNAs can bind to the SWI/SNF complex or modulate histone methylation to change chromosome structure and regulate gene expression [23,24,25]. Some lncRNAs also regulate transcription as enhancer RNAs. Enhancers are binding sites of transcription factors, and lncRNAs can bind to the site to regulate transcription [26]. Furthermore, some lncRNAs link with mRNA processing by regulation of mRNA splicing. For example, a lncRNA is known to be an important regulator in alternative splicing [27]. Moreover, many researchers have reported that lncRNAs directly or indirectly regulate the alternative splicing of downstream target genes associated with cancer development [28].

**Figure 2:**
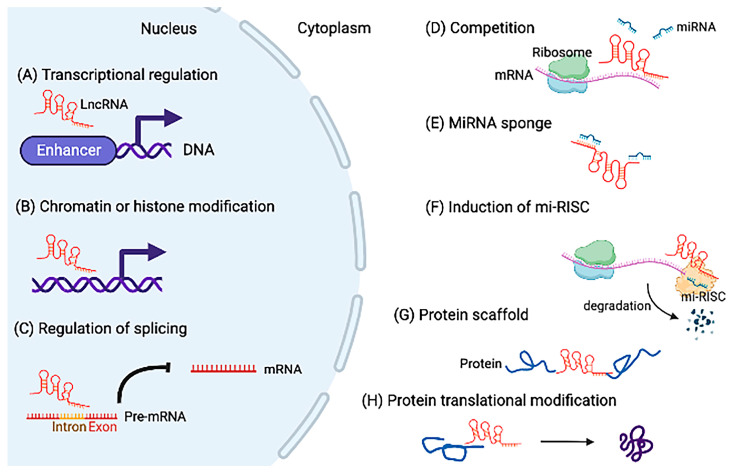
The mechanisms of long noncoding RNAs (lncRNAs)’ action in the nucleus and cytoplasm. In the nucleus: (**A**) LncRNAs act as enhancers and regulate transcription. (**B**) LncRNAs regulate chromatin or histone modification. (**C**) LncRNAs inhibit mRNA processing by the regulation of mRNA splicing. In the cytoplasm: (**D**) LncRNAs compete with miRNAs to bind mRNAs. (**E**) LncRNAs act as miRNA sponges. (**F**) LncRNAs promote the miRNA-induced silencing complex (mi-RISC) to regulate mRNA transcription. (**G**) LncRNAs act as scaffolds to modify transcriptional complexes. (**H**) LncRNAs bind to specific proteins to regulate posttranslational modification.

Similarly, lncRNAs localized in the cytoplasm can play significant roles. Some lncRNAs can act as competing endogenous RNAs (ceRNAs) against miRNA binding to target mRNAs [29]. In addition, lncRNAs can act as miRNA sponges to regulate and reduce their bioactivity [30]. Other lncRNAs can promote the miRNA-induced silencing complex (mi-RISC) to repress translation of mRNAs. LncRNAs also can act as scaffolds for some proteins to modify transcriptional or posttranscriptional complexes for regulating gene expression [31]. Other lncRNAs can bind to specific proteins to regulate translation and posttranslational modification [32,33]. We summarize the mechanisms of lncRNAs’ action in Figure 2.

### 2.3. CircRNAs

CircRNAs are generated from precursor mRNAs by back-splicing on a 5′ splice site to a 3′ splice site. Back-splicing originates from lariat-driven circularization, RNA-binding proteins (RBPs), intron pairing-driven circularization, and intron circularization. By covalent joining on the 5′ and 3′ ends, the circular structure contributes to the stability against RNA degradation and deadenylation, which gives a longer half-life compared to linear RNAs. Based on the biogenesis of circularization, circRNAs are categorized into three types: exonic circRNAs (EcircRNAs), exon-intronic circRNAs (EIcircRNAs), and intronic circRNAs (IcircRNAs).

**Figure 3:**
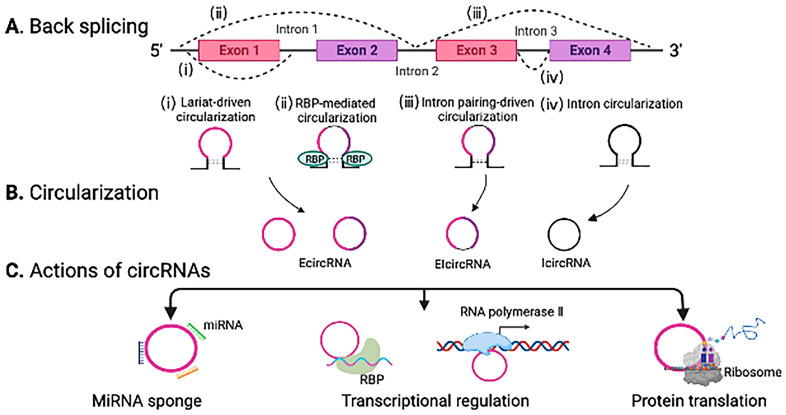
Circular RNA (circRNA) biogenesis and actions. (**A**) CircRNAs are generated by back-splicing of mRNAs on a 5′ splice site to a 3′ splice site. Back-splicing originates from (i) lariat-driven circularization, (ii) RNA-binding protein (RBP)-mediated circularization, (iii) intron pairing-driven circularization, and (iv) intron circularization. (**B**) Based on circularization, circRNAs are classified into exonic circRNAs (EcircRNAs), exon-intronic circRNAs (EIcircRNAs), and intronic circRNAs (IcircRNAs). EcircRNAs: an exon can omit splicing, leading to an intron-free transcript (from i in (**A**)). The first intron is detached and results in the 5′ splice site of exon 2 to be closer to the 3′ splice site of exon 1, leading to an ecircRNA with 2 exons (from ii in (**A**)). EIcircRNAs are generated by circularization of exons and introns (from iii in (**A**)). IcircRNAs originate from intron lariats that skip the standard intron debranching and degeneration (from iv in (**A**)). (**C**) CircRNAs can sponge miRNAs, regulate transcription by interacting with RBPs or RNA polymerase II, and encode proteins.

Accumulating evidence has demonstrated the following mechanisms of action for circRNAs. Some circRNAs can act as miRNA sponges and inhibit the activity of one or multiple miRNAs. Indeed, the circRNA sponge for miR-7 (ciRS-7) includes more than 70 conserved binding sites of miR-7 [34]. In addition, circRNAs can act as transcriptional regulators by interacting with RBPs or by binding to RNA polymerase II. For example, RBP and alternative splicing factor Muscleblind-like 1 (MBNL1) is circularized and conserved. This circRNA is strongly bound to Muscleblind-like proteins and serves as a protein scaffold or modifier during parental gene expression [35]. Furthermore, other circRNAs can be translated into proteins to regulate gene expression. For instance, circMAPK1 encodes protein MAPK1-1099a, which is known as a tumor suppressor [36]. We summarize the biogenesis and actions of circRNAs in Figure 3.

## 3. NcRNAs in Cardiac Development

### 3.1. Roles of ncRNAs in the Formation of the Heart Tube and Cardiac Morphogenesis

The heart is mainly a mesodermal derivative, although some parts of the heart, such as the cushions of the outflow tract, are constituted, in part, from the ectoderm-derived cardiac neural crest. Cardiac precursors exist in two symmetrical parts of the mesoderm lateral to the stomatopharyngeal membrane that will be differentiated into the mouth. The mesoderm is separated by the intra-embryonic coelom into a somatopleuric stratum fronting the ectoderm and a splanchnopleuric stratum opposing the endoderm. The latter stratum of the mesoderm will differentiate into the heart [37]. NcRNAs are well-established regulators of human cardiac development, specifically at a cellular level with implications for the differentiation and the specification of a diversity of cells as discussed below.

As cardiac development progresses, the precardiac mesoderm is next attached to the embryonic midline, forming an embryonic heart tube. Progressively, the embryonic heart shows the initial features of left–right asymmetric morphology by rightward looping of the premature heart tube and forthcoming embryonic ventricular and atrial chambers. Next, the atrial and ventricular chambers expand, and distinctive left and right compartments arise as a result of the development of the interatrial and interventricular septa, respectively. The final steps of cardiac morphogenesis are characterized by the achievement of atrial and ventricular septation, leading to the arrangement of a dual circuitry with distinctive systemic and pulmonary chambers [38]. Recently, understanding of the roles of several growth factor signaling cascades and transcriptional regulators in cardiac morphogenesis has increased. In addition, accumulated evidence has revealed the importance of ncRNAs in cardiac development [39]. Kay et al. identified a well-conserved miR that is involved in the development of the heart muscle by modulating WNT and TGF-β signaling pathways. They used in silico analysis to identify miR-335-3p and miR-335-5p as important regulators of cardiac morphogenesis. Using human embryonic stem cell (ESC)-derived cardiomyocytes (CMs), the study reported that modulating these miRs resulted in dysregulated key markers of cardiac differentiation. Overexpression of miR-335-3p and miR-335-5p led to increased CNX43 and TNNT2, implying the importance of WNT and TGF-β signaling pathways in the observed phenotypes [39]. Garcia-Padilla et al. also demonstrated the implication of the miR-133a/RhoA/Cdc42 axis in the early development of the posterior cardiac tube segment by regulating retinoic acid signaling [40]. In their study, the function of miR-133a as a regulator of retinoic acid signaling in heart tube formation was assessed with a variety of functional tests. These experiments involved several microinjections into subsequent cardiac precursors from primitive endocardial tubes in chick embryos. Results showed that miR-133a inhibits RhoA and Cdc42 and regulates the Raldh2/Aldh1a2 axis and selective atrial markers (Tbx5 and AMHC1), which play important roles during differentiation. This study also showed that miR-133a increases p21 levels and reduces cyclin A levels by downregulating RhoA and Cdc42, respectively, thus acting as a cell proliferation inhibitor. Moreover, retinoic acid inhibited miR-133a, concomitant with upregulated Raldh2, Tbx5, and AMHC1. This study established the existence of a negative feedback mechanism between miR-133a and retinoic acid, which is important for the early development of the posterior cardiac tube segment [40].

### 3.2. Roles of ncRNAs in Cardiac Mesoderm Specification

The NOTCH signaling pathway is regulated by the interplay between transmembrane receptors and ligands found on the membrane of adjacent cells. Four receptors (NOTCH1, NOTCH2, NOTCH3, and NOTCH4) and five ligands (JAGGED1, JAGGED2, DELTA-LIKE1, DELTA-LIKE 3, and DELTA-LIKE 4) are found in the murine and human genomes. Following their activation, a split occurs in the NOTCH receptors, and the intracellular domain of the NOTCH (NICD) is transferred to the nucleus, where it binds to the NOTCH effector RBPJ and stimulates the expression of target genes. NOTCH is involved in regulating trabeculation, compaction, and endocardial cushion development. Notably, NOTCH is crucial in ESC differentiation and controls the commitment into either the mesodermal or the neuroectodermal lineage [38]. Several ncRNAs showed synchronized expression patterns with their proximal protein-coding genes (PCGs). A set of ncRNAs transcribed in the reverse direction of their related PCGs have recently been shown as potent regulators of lineage-specifying transcription factors [41]. Kay et al. identified and characterized a lncRNA named CARdiomyocyte Maturation-Associated lncRNA (CARMA). This conserved lncRNA regulates the differentiation and maturation of CMs from ESCs in various species, including humans. The results showed that the genomic localization of CARMA is adjacent to miR-1-1HG, which is the host gene for two cardiogenic miRs (miR-1-1 and miR-133a2). A negative correlation emerged between the expression of CARMA and that of adjacent miRs, and inhibition of CARMA led to increased levels of the two miRs. The regulation of miR-133a2 by CARMA increased the expression of its direct target gene (RBPJ), which is a key effector of the NOTCH pathway. Remarkably, the study corroborated the negative association of two lncRNAs (linc1230 and linc1335), which are repressors of neuroectodermal specification, with the expression of CARMA. Furthermore, linc1230 and linc1335 were upregulated following Notch1 inhibition in ESCs. This study, therefore, indicates the presence of a network associating three novel lncRNAs, two cardiac miRs, and NOTCH signaling pathways for the organized regulation of cardiac mesoderm specification [41].

In addition, other PCGs, which are implicated in controlling lineage specification, neighbor divergent lncRNAs. This signifies the importance of noncoding transcripts in magnifying the regulatory information enclosed within genomic loci that are essential for regulating specification and differentiation. As an example, the lncRNA Fendrr is transcribed divergently from the transcription factor FOXF1. The expression of Fendrr was specifically located in the lateral plate mesoderm of the embryo and interrelated with two chromatin-modifying complexes (TrxG/MLL and PCR2) to regulate the precise expression of transcription factors involved in heart development [42]. Deletion of Fendrr in mice led to death at day 13.75 and disturbed histone modifications associated with the initiation and suppression of transcription of the above-listed transcription regulators that control cardiogenic cell fate. LncRNAs have also been shown to control gene expression by forming complexes with broadly expressed chromatin regulators and targeting their localization to specific genomic loci. In this context, Fendrr was demonstrated to couple to PRC2 and translocate it to Foxf1 and Pitx2 promoters to suppress the expression of these genes. Notably, epigenetic mechanisms similar to those initiated by Fendrr continue through various stages of differentiation, thus affecting the epigenetic landscape of cardiac development [42].

Hazra et al. showed that the dysregulation in an early embryonic lncRNA, namely the pluripotency-associated transcript 4 (Platr4), directly impacts the specification of cardiac mesoderm differentiation. The findings from their study showed that Platr4 functions as a molecular scaffold that interacts with Hippo-signaling pathway effectors (Yap and Tead4) to regulate a downstream target, Ctgf, which is required for the cardiac-lineage differentiation. Their results also showed that Platr4 deletion in mice leads to myocardial atrophy and valve mucinous degeneration associated with reduced cardiac output and heart failure. The evidence supports that Platr4 is crucial for cardiac-lineage specification and the regulation of cardiac function in mice [43].

Finally, Kim et al. identified and established a role of the lncRNA Moshe (1010001N08ik-203), which is a part of the Gata6 antisense transcripts positioned upstream of Gata6. They reported that this lncRNA is implicated not only in heart development but also in the most frequent type of congenital heart dysfunction (the atrial septal defect). The results of their study revealed that downregulation of Moshe during cardiogenesis led to Nkx2.5 inhibition in cardiac progenitor stages and caused downregulation in second heart field (SHF) lineage genes, including transcriptional factors in cardiac cells (Isl1, Hand2, and Tbx2), several endothelial genes (Cd31, Flk1, Tie1, and vWF), α-smooth muscle actin (α-SMA), and sinoatrial-node-specific genes (Shox2 and Tbx18). Kim et al. contributed to the establishment of Moshe as a key regulator of cardiac development [44].

### 3.3. Roles of ncRNAs in Embryonic CMs and Cardiac Progenitor Cells

The initial phase of identifying and characterizing ncRNAs implicated in cardiac development consisted of several genome-wide transcriptional profiling analyses to provide evidence that ncRNAs are important mechanisms of development-specific transcriptional networks. A lncRNA profile was associated with cardiac regeneration in adult zebrafish models. The methodology included the induction of heart failure in adult zebrafish by thrice incubating an anemia-inducing drug, phenylhydrazine hydrochloride, every 7 days. The drug was administered for 5 weeks, and the fish were monitored throughout a controlled regeneration period of 14 days. The results revealed that 187 lncRNAs were differentially expressed, among which 57 had human homologs. This initial investigation further contributed to supporting the implication of ncRNAs in cardiac development [45]. Moreover, several other studies involved attempts to establish the cardiac lncRNA profiles associated with heart development in vivo. Early and groundbreaking research investigating the mechanism underlying the association between chromatin structure and gene expression in cardiac commitment revealed that many ncRNAs are differentially and dynamically expressed [46]. This study used both in vitro and in vivo models of cardiogenesis to assess this landscape at distinct cell stages, including ESCs, mesodermal cells, cardiac progenitor cells, and CMs. Results revealed that hundreds of ncRNAs were differentially expressed in a stage-specific and dynamic manner. Intriguingly, several ncRNA expression patterns were associated with their adjacent promoters of PCGs, indicative of potential cis-regulatory roles throughout the differentiation of CMs. Similarly, Li et al. conducted RNA sequencing on mouse hearts after 8 weeks of controlled exercise-induced hypertrophy and after transverse aortic constriction (TAC) for 2 or 8 weeks to induce pathological hypertrophy or heart failure. They identified potential lncRNAs that were differentially expressed and significantly associated with each phenotype. Their results revealed an exercise-regulated cardiac lncRNA that was named lncExACTs [47]. This lncRNA was evolutionarily conserved and was reduced in exercised hearts despite augmented expression in human and experimental models of heart failure. Their results also showed that overexpression of lncExACT1 initiated pathological hypertrophy and heart failure. Conversely, its inhibition provoked physiological hypertrophy, stimulating a protective mechanism against cardiac fibrosis. The mechanism of lncExACT1 action involved the regulation of miR-222, calcineurin, and Hippo/Yap1 signaling pathways by altering DCHS2. DCHS2 overexpression in CMs caused uncontrolled hypertrophy and reduced cardiac regeneration in zebrafish, leading to increased scarring after injury. In contrast, DCHS2 inhibition in a mouse model led to controlled hypertrophy. This study, therefore, established the novel lncExACT1/DCHS2 pathway as an important regulator of cardiac development [47].

## 4. NcRNAs in Cardiovascular Diseases

### 4.1. Atherosclerosis

Atherosclerosis is one of the leading causes of metabolic syndrome, which is a trigger for CVDs. Many molecular mechanisms are implicated in the pathogenesis and development of atherosclerosis. Chronic stimulations, such as high blood pressure and plaque accumulation on the arterial walls, induce inflammatory responses and pathological changes in the walls. The pathological changes include angiogenesis, lipid dysregulation, cell proliferation, and apoptosis. The arterial walls consist of three layers. The most internal layer, tunica intima, consists of endothelial cells. The middle layer, tunica media, is composed of smooth muscle cells and elastic tissues. The most external layer, tunica externa, is made up of an external elastic membrane, connective tissues, and fibroblasts. In the early stage of atherosclerosis, stimulations such as blood pressure and lipid accumulation trigger endothelial injury, leading to endothelial dysfunction. The process causes the migration of inflammatory cells, especially macrophages and T cells, to the arterial walls, followed by the proliferation of vascular smooth muscle cells (VSMCs) [48]. VSMCs, which are abundant in tunica media, regulate vasoconstriction and vasodilation to maintain appropriate hemodynamics in response to external and internal stimulation in the body. However, alternation and imbalance of vascular structure cause dysregulation of vascular tone and impair endothelial function [49,50].

Interestingly, VSMCs have phenotypic plasticity and are not terminally differentiated. In response to local stimulation or damage, VSMCs shift their roles from muscle contraction to protein synthesis for proliferation and migration [51]. VSMCs are also known to exhibit characteristics of osteoblasts, adipocytes, and macrophage-derived foam cells. The phenotype shift in VSMCs from contraction type into osteogenic type causes vessel calcification [52]. In addition, VSMC apoptosis is associated with plaque rupture, coagulation, and vessel remodeling. Accumulating evidence indicates that VSMC apoptosis accelerates vascular remodeling and the progression of atherosclerosis [53].

Zhang et al. showed that lncRNA X-inactive specific transcript (XIST) regulates proliferation and migration in oxidized low-density lipoprotein (ox-LDL)-stimulated VSMCs by miR-539-5p sponging [54]. The authors found that lncRNA XIST was increased in ox-LDL-treated VSMCs, and injection of the small hairpin (sh)-XIST to atherosclerotic mice fed with a high-fat diet (HFD) significantly reduced atherosclerotic plaques. In addition, they showed that XIST knockdown markedly attenuated cell migration and significantly decreased proliferation-related markers, including proliferating cell nuclear antigen (PCNA) and Ki-67, and migration-related proteins, including matrix metalloproteinase-2 (MMP-2) and MMP-9. Mechanistically, Zhang et al. verified that lncRNA XIST bound to miR-539-5p and that miR-539-5p bound to apoptotic secreted phosphoprotein 1 (SPP1). Finally, they demonstrated that the gain of miR-539-5p decreased proliferation and migration in ox-LDL-treated VSMCs via suppression of SPP1. In conclusion, their study showed that suppression of lncRNA XIST attenuated the differentiation of VSMCs in atherosclerosis by regulating the miR-539-5p/SPP1 axis.

Lin et al. reported the association between mitochondrial dynamic-related lncRNA (MDRL) and atherosclerosis in VSMCs [55]. They first demonstrated that MDRL is downregulated in atherosclerotic plaques, and overexpression of MDRL ameliorated the burden of plaques in the aortic roots of low-density lipoprotein receptor knockout (LDLR^−/−^) mice. In addition, they reported that miR-361 mimic injection inhibited the regression of plaques in the MDRL-treated LDLR^−/−^ mice. To investigate the mechanism, they used luciferase reporter analysis and showed that miR-361 bound to 3′UTR of sequestosome 1 (SQSTM1), which regulates the NLR family pyrin domain containing 3 (NLRP3) inflammasomes. MiR-361 overexpression also induced apoptosis and inflammation in VSMCs. Overall, this study suggested that the lncRNA MDRL/miR-361/SQSTM1 axis in VSMCs plays an important role in the regulation of atherosclerotic progression.

Another type of ncRNA, circRNAs, have also been reported to be implicated in the regulation of atherosclerosis. For example, Zhang et al. demonstrated the roles of hsa_circ_0086296 in atherosclerosis [56]. They showed that circ_0086296 is upregulated in human carotid artery plaques, ox-LDL-treated human umbilical vein endothelial cells (HUVECs), and plaques in the aorta of atherosclerotic mice. Loss- or gain-of-functional studies of circ_0086296 in ox-LDL-treated HUVECs and apolipoprotein E knockout (ApoE^−/−^) mice fed with HFD showed that circ_0086296 regulated endothelial proliferation and migration as well as inflammatory damage. Mechanistically, they showed that circ_0086296 sponges miR-576-3p, leading to upregulation of interferon-induced proteins associated with tetratricopeptide repeats 1 (IFIT1) and signal transducer and activator of transcription 1 (STAT1) signaling. These results suggested that circ_0086296 plays important roles in the regulation of atherosclerosis progression via the miR-576-3p/IFIT1/STAT1 axis.

In addition, Lin et al. reported that circ_0021155 was associated with the phenotypic transformation of VSMCs [57]. They found that circ_0021155 was related to transient receptor potential cation channel subfamily m member 7 (TRPM7), which regulates the proliferation and migration of VSMCs in atherosclerosis [58]. Lin et al. found that circ_0021155 was upregulated in ox-LDL-treated human aorta vascular smooth muscle cells (HASMCs), and overexpression of circ_0021155 promoted HASMC proliferation and migration with a decrease in the protein levels of α-actin, calponin, and smooth muscle myosin heavy chain (SMMHC) as well as an increase in TRPM7 protein levels. Furthermore, they showed that circ_0021155 downregulated miR-4459, and miR-4459 negatively regulated HASMC proliferation and migration. The ceRNA mechanism of the circ_0021155/miR-4459 axis was verified via dual luciferase assays. In conclusion, their study suggested that the circ_0021155/miR-4459/TRPM7 axis regulated the phenotypic transformation of VSMCs in atherosclerosis. Similar mechanisms of action have been observed in a diversity of ncRNAs, which are shown in recent studies published in 2022–2023 (Table 1).

### 4.2. Cardiac Arrhythmia

Cardiac arrhythmias are a heterogenic set of dysfunctions in the heart rhythm. The physiological dysfunctions linked to specific arrhythmias are extremely complex, and rhythm-associated ion channels implicated in the initiation or progression of the action potential are well documented. NcRNAs have been shown to regulate a variety of ion channels and intercellular connection proteins, including connexins. In this section, we discuss recent findings on the implication of ncRNAs in the development of the most common forms of arrhythmias with a focus on therapeutic and clinical relevance.

#### 4.2.1. Atrial Fibrillation

Atrial fibrillation (AF) is an irregular heart rhythm, which generally leads to heart palpitations and asthenia. It is the most recurrent form of arrhythmia worldwide. Xie et al. used clinical data to identify potential lncRNAs that are associated with the development of AF. They used a set of functional tests on atrial appendage samples from the Gene Expression Omnibus database. Their results showed that six lncRNAs (RP11-532N4.2, LINC00844, RP3-332B22.1, UNC5B-AS1, RP11-557H15.4, and RP11-432J24.5) were differentially expressed in AF patients compared to normal subjects. The signaling pathways associated with these lncRNAs were the calcium signaling pathway and toll-like receptor signaling pathway. Xie et al. also identified immunological signaling pathways in AF patients that were significantly associated with the identified lncRNAs [83].

The dysregulation of tissue blockers of matrix metalloproteinases (TIMPs)/MMPs linked to collagen upregulation is a key factor in the development of AF. The level of miR-146b-5p, which is a direct inhibitor of TIMPs, was increased in atrial CMs following AF. Ye et al. demonstrated that miR-146b-5p regulates TIMP4, contributing to the initiation and progression of atrial fibrosis in AF. Using human induced pluripotent stem cell-derived atrial cardiomyocytes (hiPSC-aCMs) with miR-146b-5p inhibitors and an animal model of myocardial infarction, Ye et al. showed that downregulation of miR-146b-5p was associated with positive outcomes on atrial fibrosis. Furthermore, increased expression of this miR took place in the fibrotic atrium of canines with AF concurrent with decreased expression of TIMP4. Some profibrotic markers, such as MMP9, TGFβ1, and COL1A1, were downregulated upon miR-146b-5p knockdown, whereas TIMP4 caused inversed expression patterns. The results from their study established the importance of the miR-146b-5p/TIMP4 axis in cardiac fibrosis during AF [84].

Another mechanism underlying AF is ferroptosis, which is defined as iron-dependent cell death associated with an extreme buildup of peroxidized polyunsaturated fatty acids. Liu et al. used a rapid pacing model in vitro and a canine model of rapid pacing to establish the implication of miR-23a-3p in ferroptosis during AF. Their results revealed an elevation in the concentration of malondialdehyde and total ions in atrial tissues from the pacing groups. This increase was associated with increased levels of proinflammatory markers, leading to electrophysiological remodeling. Results also demonstrated that SLC7A11 is a direct target of miR-23a-3p and that the direct regulation of this gene was associated with ferroptosis because prooxidative markers and proteins implicated in ferroptosis, including FTH1 and GPX4, were dysregulated with a positive association with the development of AF [85].

Another miR-dependent gene regulation elicits the initiation and progression of AF. A two-pore-domain potassium channel, tandem of P domains in a fragile inner repairing K+ channel-linked acid-sensitive K+ channel 1 (TASK-1) is an atrial-specific ion channel and is increased in AF. TASK-1 knockdown extends the atrial action potential length to comparable intensities as in patients with sinus rhythm. In a study, a set of miRs were tested for their potential to regulate KCNK3 and TASK-1 in vitro. Among those miRs, miR-34a upregulated TASK-1 and current and promoted a reduction in the resting membrane potential of Xenopus laevis oocytes that express hTASK-1. The study also used clinical samples to demonstrate that this miR was increased in the atrial tissues of AF patients. The results from this study imply an essential pathophysiological connection between miR-34a and AF progression by regulating the TASK-1 potassium channel [86].

#### 4.2.2. Bradyarrhythmia

Bradyarrhythmia (BA), also known as bradycardia, is a heart dysfunction characterized by a decreased heart rate with values generally lower than 60 beats/min. The main etiological factors of BA include the dysregulation of sinus, atrial or junctional bradycardia, and a complex transmission system (atrioventricular block). The most recurrent form of BA is asymptomatic bradycardia, which usually occurs in trained athletes or during sleep [87]. The development of BA is regulated at a molecular level by a variety of molecules, including ncRNAs. Yanni et al. used an animal model of heart failure that developed sinus bradycardia to demonstrate the function of miR-370-3p in regulating the development of BA [88]. Their results showed increased expression of miR-370-3p in the group of mice with sinus bradycardia. These animals also exhibited reduced pacemaker channels and decreased protein levels of HCNA with an associated reduction in the related ionic current in the sinus node. Their results also revealed that HCN4 is a direct functional target of miR-370-3p and that miR-370-3p directly binds to HCN4 mRNA to repress its activity. The injection of anti-miR-370-3p in mice after inducing heart failure led to reduced miR-370-3p expression, increased HCN4 mRNA levels, and increased ionic current in the sinus node, leading to a decreased sinus bradycardia. Overall, Yanni et al. showed the functional impact of the miR-370-3p/HCNA axis in regulating BA [88].

Transcription factors also control the expression of genes involved in BA by modulating a variety of effectors including miRs. Aminu et al. demonstrated the implication of miRs in the regulation of ion channels in the sinus node. By using RNA-seq and bioinformatics, they assessed the expression profile and projected interaction among major cell markers [89]. Their results showed that miR-486-3p was differentially expressed in the adult human sinus node vs. right atrial tissue. The results also revealed that miR-486-3p repressed HCN4 and that this interaction can be used to manage sinus node dysregulation such as BA [89].

#### 4.2.3. Ventricular Arrhythmias

Ventricular arrhythmias (VA) are defined as irregular heartbeats that derive from the ventricles. These types of arrhythmias are characterized by increased heart rate, causing decreased circulation of oxygen-rich blood to the body, which may result in cardiac arrest. The primary cause of VA is sympathetic remodeling that originates in myocardial infarction (MI), leading to abrupt cardiac death. NcRNAs are important regulators of inflammation and sympathetic remodeling following MI. Li et al. identified and characterized lncRNAs that are potentially implicated in VA following MI [90]. In their study, differentially expressed lncRNAs were identified by RNA-seq on nonactivated M0- and proinflammatory M1-type macrophages. The results demonstrated that the lncRNA LOC100911717 (LOC10) was increased in infarcted hearts and M1-type macrophages, but not in M0-type macrophages. In addition, RNA pull-down assays demonstrated that LOC10 might interact with growth-associated protein 43 (GAP43). The results further supported the reduction in GAP43 expression and in VA incidence after LOC10 knockdown in rat hearts following adeno-associated virus (AAV) injection [90]. This study demonstrated the role of the LOC10/GAP43 axis in the regulation of VA.

Another critical factor of VA-mediated heart failure is NLRP3 inflammasomes. Liang et al. recently demonstrated the role of a lncRNA (SOX2-overlapping transcripts (SOX2-OT)) in regulating NLRP3 inflammasome-mediated VA. They found that the levels of SOX2-OT and NLRP3 inflammasomes are significantly increased after VA. SOX2-OT inhibition led to decreased NLRP3 concurrent with increased miR-2355-3p expression. The deletion of SOX2-OT caused downregulation of proinflammatory and stress markers. Their study showed that profibrotic effectors were decreased after SOX2-OT downregulation. Liang et al. thus showed a key role of the SOX2-OT/miR-2355-3p/NLRP3 axis in regulating VA in mice [91]. Moreover, Shi et al. demonstrated the functional role of miR-1231 in regulating L-calcium in VA using a chronic model of heart failure [92].

#### 4.2.4. Tachycardia

Tachycardia (TrA), known to be characterized by a fast heartbeat, is also regulated at a molecular level. The implication of ncRNAs in the development and progression of this pathology has been a recent scientific focus. Djalinac et al. associated microarray analysis with an in vitro model of isometric stretch (continual tachycardia at 2.5 Hz in human atrial trabeculae) and identified ncRNAs associated with TrA. Their results revealed that the expression of miR-1183 was significantly upregulated in TrA. The in silico analysis revealed that ADAM20 and PLA2G7 were potential targets of miR-1183. Data obtained from human samples further showed decreased levels of ADAM20 and PLA2G7 following TrA concurrent with increased miR-1183. These findings suggest the importance of miR-1183 in the regulation of TrA [93].

TrAs are generally categorized by the chamber from which they derive. Supraventricular arrhythmia (SA) is an example of TrA that starts in the upper compartments of the heart (the atria). Park et al. recently showed that specific miRNAs identified in urine samples from humans might regulate SA by inhibiting the phosphorylation of key proteins involved in calcium handling following SA. Transcriptomic data from the urine sample revealed significantly reduced levels of seven miRNAs, including miR-3613, miR-6763, miR-423, miR-3162, miR-1180, miR-6511, and miR-3197. The changes in these miRs were associated with increased expression of profibrotic markers, such as Col I, Col III, fibronectin, and TGF-β. MiR-423 specifically was shown to regulate calcium-handling proteins, such as the phosphorylated calmodulin-dependent protein kinase II [94]. The results from this study highlight the significance of miRs capable of regulating calcium-handling proteins in the management of TrAs.

### 4.3. Cardiac Fibrosis

Cardiac fibrosis is characterized by the pathological accumulation of collagen and other extracellular matrix (ECM) proteins produced by fibroblasts in the heart. Although fibrosis is a fundamental healing response to acute cardiac injury, excessive or pathological fibrosis can impair cardiac function and contribute to the development of various cardiac diseases because the reparative capacity of CMs is limited. Lower levels of cardiac fibrosis are clinically associated with drug-induced reverse remodeling for heart failure [95]. Identifying the underlying mechanisms of fibrosis and developing effective strategies to prevent or reverse fibrosis at an early stage are, therefore, urgently needed for heart failure.

Jinghua et al. demonstrated that the TDRG1/miR-605-3p/TNFRSF21 axis is involved in the modulation of fibrogenesis and inflammatory response in human cardiac fibroblasts (HCFs) stimulated with TGF-β1. Specifically, the lncRNA TDRG1 was upregulated in TGF-β1-stimulated HCFs, and its knockdown inhibited fibrogenesis and inflammatory response in TGF-β1-stimulated HCFs. Notably, TNFRSF21 was identified as a target of miR-605-3p, and TNFRSF21 reversed the effects of TDRG1 knockdown, thereby exacerbating fibrogenesis and inflammatory responses in TGF-β1-stimulated HCFs [96]. Quaife et al. also conducted experiments in HCFs stimulated with TGF-β1. They discovered that LINC01013, which is associated with small open reading frames (smORFs), is upregulated following TGF-β1 stimulation of HCFs. This upregulation led to the production of a biologically active micropeptide. Knockdown of LINC01013 reduced baseline markers of fibroblast activation and dampened the response to TGF-β1. However, overexpression of LINC01013ORF induced markers of fibroblast activation to the same level as observed with TGF-β1 stimulation. Although the exact mechanism by which this micropeptide affects fibrosis has yet to be elucidated, its localization suggests that mitochondrial metabolism may play a role [97]. In addition, Chingnon et al. reported that LINC01013 may induce calcification of the aortic valve via the TGF-β/CCN2/CTGF axis [98].

As noted above, various pathological conditions can cause cardiac fibrosis. Feng et al. investigated the involvement of miR-9 in diabetic cardiomyopathy and showed that miR-9 suppresses the production of ECM proteins and inflammatory molecules in human cardiac microvascular endothelial cells (HCMECs) and mouse cardiac endothelial cells (MCECs) upon glucose loading. MiR-9 transgenic (TG) mice also showed inhibition of diabetes-induced myocardial fibrosis. Furthermore, they proposed a novel pathway by which lncRNA ZFAS1 regulates miR-9 and exerts its effects on glucose via the PRC2 complex [99]. Peng et al. studied the lncRNA Airn in genetically engineered mice with intramyocardial AAV injection, showing that Airn suppresses myocardial fibrosis in diabetic cardiomyopathy via the IMP2/p53 axis in an m6A-dependent manner [100]. Zhou et al. performed a comprehensive analysis in a cardiac fibrosis model and identified lncRNA THBS1-AS1 as a regulator of TGFBR1 through miR-221/222 sponge activity. Furthermore, under TGF-β1 stimulation, forced expression of miR-221/222 or knockdown of TGFBR1 significantly reversed cardiac fibroblast activation induced by THBS1-AS1 overexpression. In vivo, specific knockdown of THBS1-AS1 in activated cardiac fibroblasts significantly reduced TAC-induced cardiac fibrosis in mice [101].

Cardiac fibrosis has been observed not only in the ventricles but also in the atria. Tan et al. specifically studied atrial fibrosis and AF. They found that HOTAIR enhances the stability of Wnt5a by recruiting PTBP1. Wnt5a overexpression reversed the inhibition of proliferation, migration, and fibrosis mediated by HOTAIR silencing in Ang II-stimulated primary atrial fibroblasts. In an in vivo model, Ang II significantly promoted fibrosis and structural disorder in myocardial tissues, whereas this phenomenon was significantly mitigated by HOTAIR knockdown. Therefore, HOTAIR could promote myocardial fibrosis in AF by binding with PTBP1 to increase Wnt5a stability and activate the ERK/JNK signaling pathway. Tan et al. concluded that this finding could be applied to clinical AF patients [102].

### 4.4. Cardiac Hypertrophy

Cardiac hypertrophy is a hypertrophic change of CMs against wall stress. Chronic pressure overload or neurohormonal stimulation induces CM hypertrophy to reduce the wall stress and maintain cardiac contraction [103]. However, the compensatory remodeling of left ventricular hypertrophy causes numerous problems, such as reduced left ventricle volume, diastolic dysfunction, and frequent cardiac arrhythmia, which lead to chronic heart failure. The hypertrophic mechanism is accompanied by alternations of many cellular signaling pathways, cellular metabolisms, fetal gene programs, and mitochondrial function [104]. More importantly, many studies have demonstrated that ncRNAs are implicated in the molecular mechanisms of hypertrophic processes.

Tu et al. showed that lncRNA terminal differentiation-induced noncoding RNA (TINCR) has a pivotal role in cardiac hypertrophy [105]. They showed that TINCR was downregulated and that miR-211-3p was upregulated in TAC-induced mouse hearts and angiotensin II (Ang II)-treated H9C2 cells. Furthermore, they demonstrated that knockdown of miR-211-3p alleviated cardiac hypertrophy both in vitro and in vivo. In addition, using dual luciferase and RNA immunoprecipitation (RIP) assays, they revealed that TINCR directly binds to miR-211-3p. Overexpression of TINCR also suppressed TAC-induced cardiac hypertrophy. Mechanistically, miR-211-3p directly targeted vascular endothelial growth factor B (VEGFB) and thus regulated the levels of stromal cell-derived factor-1α (SDF-1α) and C-X-C chemokine receptor type 4 (CXCR4). CXCR4 is known to suppress cardiac hypertrophy [106]. Thus, they showed that miR-211-3p plays an important role in the regulation of hypertrophic process via the VEGFB/SDF-1α/CXCR4 pathway.

Another group reported that miR-30d reversed cardiac hypertrophic remodeling [107]. Yan et al. showed a decrease in miR-30d in the serum of patients with chronic heart failure and in in vivo and in vitro hypertrophic models. Phenylephrine (PE)- and Ang II- treated neonatal rat ventricular cardiomyocyte (NRVC) studies showed that gain of miR-30d ameliorated cardiac hypertrophy, whereas the opposite phenotype was observed by loss of miR-30d. The protective role of miR-30d against cardiac hypertrophy was consistent with in vivo experiments using miR-30d TG rats injected with isoproterenol (ISO). Mechanistically, methyltransferase enhancer of zeste homolog 2 (EZH2) promoted H3K27me3 methylation in the promoter region of miR-30d and suppressed its expression. They also showed that mitogen-activated protein kinase kinase kinase kinase 4 (MAP4K4) and glucose-regulated protein 78 (GRP78), which inhibit prohypertrophic nuclear factor of activated T-cell (NFAT), are direct and functional targets of miR-30d. Finally, mice injected with AAV9 for miR-30d at 2 weeks after TAC reversed hypertrophic remodeling post-TAC. In conclusion, miR-30d regulated cardiac hypertrophy by regulating the MAP4K4/GRP78 a/NFAT pathway.

CircRNAs also have been reported to be implicated in the regulation of cardiac hypertrophy. For example, Lin et al. found that circ_0001006 has an important role in cardiac hypertrophy [108]. They showed that circ_0001006 was upregulated in TAC-induced mouse hearts and neonatal rat ventricular cardiomyocytes (NRVCs) treated with Ang II. The authors demonstrated that miR-214-3p bound to circ_0001006 and that miR-214-3p interacted with 3′UTRs of serine/threonine-protein kinase PAK 6 (PAK6). In addition, the gain of circ_0001006 increased cell size, which was abolished by miR-214-3p mimic or small interfering (si)-PAK6. Finally, they showed that AAV9 sh-circ_0001006-injected mice significantly attenuated cardiac hypertrophy in TAC mice as well as decreased the markers of cardiac hypertrophy and heart failure. In conclusion, the authors demonstrated that circRNA_0001006 exacerbated cardiac hypertrophy via the miR-214-3p/PAK6 axis. Other recent studies supporting the implication of ncRNAs in cardiac hypertrophy and heart failure are summarized in Table 2.

### 4.5. Myocardial Infarction

Myocardial infarction (MI) is a pathological condition that disrupts cardiac function by inducing myocardial ischemia (e.g., occlusion of the coronary artery). In the United States alone, 605,000 new cases of MI occur each year, with an additional 200,000 cases of second or subsequent MI. It is considered one of the primary causes of mortality worldwide [1]. Moreover, about 20% of these cases remain asymptomatic without exhibiting chest pain or other related symptoms [1]. In addition, coronary artery disease (CAD) is considered the primary cause of heart failure with an estimated incidence of 20% [1]. Although the pathogenesis of MI remains incompletely understood, an increasing body of research suggests that ncRNAs may be involved in its development and progression. Given that minimizing the duration of coronary revascularization therapy during MI leads to reduced mortality, ncRNAs are currently undergoing extensive investigation as potential diagnostic tools in the clinical field. Recently, Li et al. reported a comprehensive analysis of circRNAs in the peripheral blood of 80 acute myocardial infarction (AMI) patients before percutaneous coronary intervention (PCI) surgery. CircTMEM165, circUBAC2, circZNF609, circANKRD12, and circSLC8A1 were useful diagnostic biomarkers, and circSLC8A1 was associated with CM apoptosis [123].

Yang et al. investigated the use of ncRNAs to predict patients with ST-segment elevated myocardial infarction (STEMI), who did not achieve flow because of microvascular obstruction or other causes after primary PCI. The results showed that MALAT1, miR-30e, and miR-126 were candidate ncRNAs and that MALAT1 functioned as a sponge for miR-30e and miR-126. The authors concluded that MALAT1 is clinically useful for predicting no flow [124]. The usefulness of miRs for diagnosis and prognosis was also reported by Elbaz et al., who conducted a case–control study of 67 patients with MI and 80 patients with high vascular risk but without known CAD. The study revealed that miR-223 and miR-186 correlated with long-term prognosis after adjustment for left ventricular ejection fraction (LVEF) [125]. The diagnostic potential of lncRNA MALAT1 in the clinical setting has also been reported [126]. Liu et al. investigated the effect of MALAT1 on cardiac ischemia-reperfusion (I/R) injury. They showed that MALAT1 inhibits the activation of the IGF1R/PI3K/AKT/eNOS axis by binding to miR-133a-3p, thereby causing inhibition of cell survival and promotion of apoptosis in CMs under I/R conditions [127]. Nugroho et al. demonstrated that miR-411, which is more highly expressed in neonatal rat CMs than in adult rat CMs, plays a significant role in promoting CM proliferation. Direct injection of miR-411 into the rat myocardium promoted cell proliferation and reduced apoptotic cells, thereby preventing cardiac dysfunction mediated by MI. Furthermore, in vitro experiments using H9C2 cells showed that miR-411 modulates the Hippo/YAP pathway and is involved in both cell survival and proliferation [128].

Notably, significant changes occur in cellular composition within the heart during MI. We recently used innovative mouse models and showed that lncRNA MIAT exacerbates detrimental post-MI remodeling and that miR-150 mitigates excessive detrimental post-MI remodeling mediated by MIAT. This effect was achieved by blunting cardiac fibroblast activation via direct functional inhibition of HOXA4. In addition, we showed that miR-150 directly and functionally inhibits profibrotic HOXA4 in HCFs, leading to protective effects [129]. NcRNAs play important roles not only in the heart but also in communication with other organs. One group focused on the poor prognosis of heart failure patients affected by muscle disuse atrophy. They investigated miRs present in blood exosomes by inducing disuse atrophy in an I/R mouse model. Findings indicated that miR-16-5p, which is secreted into exosomes because of muscle atrophy, promotes CM apoptosis via the SESN1/mTOR axis, thereby deteriorating cardiac function [130]. Additional recent studies supporting the significance of ncRNAs in MI and CAD are summarized in Table 3.

### 4.6. Pulmonary Hypertension

Pulmonary hypertension (PH) encompasses a heterogeneous group of disorders characterized by elevated pulmonary arterial pressure, which, if left untreated, can lead to heart failure. In the latest guidelines, PH is classified into five groups based on pathophysiologic mechanisms, clinical presentation, hemodynamic characteristics, and therapeutic management: (1) pulmonary arterial hypertension (PAH), (2) PH due to left heart disease, (3) PH due to lung disease and/or hypoxia, (4) chronic thromboembolic pulmonary hypertension (CTEPH), and (5) PH with unclear multifactorial mechanisms [145]. The complex etiology of PH is characterized by dysfunctional proliferation and migration of pulmonary endothelial cells (PECs), pulmonary arterial smooth muscle cells (PASMCs), and plexiform lesion formation. This progressive remodeling of pulmonary arteries leads to the narrowing of the arterial lumen and increases the mean pulmonary arterial pressure (mPAP) and pulmonary vascular resistance (PVR) [146].

Numerous studies have shown the crucial roles of miRs and lncRNAs in the pathogenesis of pulmonary vascular remodeling as evidenced in various forms of PH. Li et al. used dihydroartemisinin (DHA)-treated in vivo and in vitro PH models to show that the protective effects of DHA are conveyed through miR regulation [147]. Their study showed that miR-335 increased PASMC proliferation and migration and that treatment with DHA downregulated miR-335 expression and improved vascular remodeling. Using dual-luciferase reporter assays, the authors showed that VANGL2 was a direct target of miR-335, and the injection of miR-335 inhibitor in PASMCs with VANGL2 knockdown failed to improve adverse proliferation and migration. They further showed that the protective effects of DHA are reversed by miR-335 upregulation. This study demonstrated that DHA prevents PH through the regulation of hypoxia-induced miR-335 expression and suppresses VANGL2 downregulation. Interestingly, another study by Cai et al. showed that DHA exerts the same therapeutic effects by upregulating ELAVL2, which positively regulates miR-503 [148]. QRT-PCR analysis showed that decreased miR-503 expression under hypoxic conditions was reversed with DHA treatment and that injection of miR-503 inhibitor reversed the protective effects of DHA. The authors identified ELAVL2 as a key RBP that is expressed at low levels under hypoxic conditions. They showed that ELAVL2 knockdown caused increased hypoxia-induced proliferation and migration of PASMCs and downregulation of miR-503 expression. This study established the protective effects of DHA in PH through the ELAVL2/miR-503/PI3K/AKT axis and provided a potential therapeutic target for the treatment of PH. Wang et al. found that elevated miR-27b-3p induced glioma-associated oncogene homolog 1 (GLI1) expression in endothelin-1 (ET-1)-treated PASMCs [149]. Their study showed elevated levels of GLI1 in ET-1-treated PASMCs, whereas knockdown of GLI1 decreased PASMC proliferation and migration mediated by ET-1. GANT58, a GLI1 inhibitor, suppressed pulmonary vascular remodeling, demonstrating the role of GLI1 in PAH development. F-box and WD repeat domain-containing protein 7 (FBXW7) was shown to play a role in the pathogenesis of PH. Krupel-like factor 5 (KLF5), a transcription factor regulating diverse cellular processes, played a role in PASMC proliferation and apoptotic resistance in PAH. The authors showed that elevated levels of miR-27b-3p negatively regulated FBXW7 expression in PASMCs, upregulated KLF5, and induced GLI1 expression. This study showed the critical role of GLI1 in PAH development and provided a potential target for treatment through the FBXW7/KLF5/GLI1 pathway.

CircRNAs have also been reported to be implicated in the development and progression of PH. Sun et al. found that circular RNA-g-secretase-activating protein (circGSAP) has a suppressive role in the progression of ischemic pulmonary arterial hypertension (IPAH) via upregulation of BMPR2, a key mediator of vascular homeostasis in IPAH [150]. They found that circGSAP expression was downregulated in patients with IPAH and that circGSAP overexpression reduced proliferation and migration as well as increased cell mortality of PECs in both normoxic and hypoxic conditions. In addition, they found that circGSAP bound to miR-27a-3p and suppressed the regulatory effects on its downstream target BMPR2, thus increasing BMPR2 expression and regulating PEC’s dysfunction.

Fibroblast growth factor 21 (FGF21) has been reported to have a cardioprotective role in the progression of CVDs [151]. Li et al. showed that FGF21 promotes the expression of H19, a lncRNA in PASMCs [152]. They found that FGF21 treatment significantly alleviated PH in hypoxia-exposed mice. In addition, they analyzed the expression of lncRNAs and found marked upregulation of H19 in FGF21-treated mice subjected to hypoxia. Li et al. also showed that H19 inhibited the mTORC1/EIF4EBP1 axis, suppressed mRNA translation, as well as disrupted cell survival, migration, and proliferation. Their study illustrates the potential use of FGF21 and H19 as a therapeutic strategy in PH.

Jiang et al. explored lncRNA SRY-box transcription factor 2 overlapping transcript (SOX2-OT) in PAH and found that elevated SOX-OT levels served as a potential diagnostic marker for PAH [153]. They found that inhibiting SOX2-OT expression in hPASMCs has the potential to reverse hypoxia-induced proliferation, migration, antiapoptosis, and inflammation by modulating the miR-455-3p/SUMO1 axis. Serum SOX2-OT levels were highly expressed in PAH patients with levels distinctly different from healthy controls. This study suggests that SOX2-OT serves as a valuable diagnostic biomarker and target for therapeutic strategies for PAH. Further recent studies supporting the significance of ncRNAs in pulmonary remodeling are summarized in Table 4.

## 5. Summary and Conclusions

In the most up-to-date research articles, we find that there are common ncRNAs for several CVDs, indicating that these ncRNAs are potential novel therapeutic targets to be considered for CVDs. For example, miR-21, miR-27a-3p, miR-27b-3p, and miR-214-3p play key roles in CH (Table 2) and PH (Table 4). MiR-126 is also known to regulate MI (Table 3) and PH (Table 4), and miR-335 controls AS (Table 1) and PH (Table 4). Moreover, lncRNA H19 plays vital roles in AS (Table 1), CH (Table 2), and PH (Table 4). It has been shown that two lncRNAs, MIAT and MALAT1, are key regulators for CH (Table 2) and MI (Table 3). Last, lncRNA PVT1 modulates the pathogenesis of AS (Table 1) and MI (Table 3).

With the molecular diagnosis of CVDs via the most prevalent gene panels, considerable progress has been made in the fields of cardiac development and CVD. Despite this advance, the causes of CVDs remain unresolved in a substantial percentage of patients. NcRNAs have emerged as promising targets for early diagnosis of and therapeutic development for CVDs because many recent studies have provided novel insights into their impact on various developmental stages and disease states. For instance, the defects in cardiac development and the progression of CVDs are followed by dynamic dysregulation of the ncRNA regulatory network. NcRNAs have also been shown to play key roles in a variety of biological processes that contribute to maintaining cardiovascular function. However, an urgent need still exists to better understand the biology and actions of ncRNAs when exploring their therapeutic effects in patients. Most preclinical studies also remain untranslated to clinical stages. The new insights on regulatory mechanisms of ncRNAs in the future could facilitate the scientific community’s defining novel approaches for the management of abnormalities of cardiac development and CVDs.

## Figures and Tables

**Table 1:** Noncoding RNAs in atherosclerosis (AS).

NcRNA	Expression in AS	Experimental Models	Mechanisms of Action	Roles	Reference
MiR-499-5p	↑	Oxidized low density lipoprotein (ox-LDL)-treated mouse aortic vascular smooth muscle cells (MAVSMCs) and apolipoprotein E knockout (ApoE^−/−^) mice	Regulation of SOX6	Proliferation and migration of smooth muscle cells	[59]
MiR-32-5p	↑	Ox-LDL-treated human umbilical vein endothelial cells (HUVECs)	Regulation of AIDA	Inflammation	[60]
MiR-351	↑	Ox-LDL-treated mouse aortic endothelial cells and miR-351 knockout mice	ITGB3/PIK3R1/AKT pathway	Apoptosis, lipid accumulation, and oxidative stress	[61]
MiR-130a-5p	↓	Ox-LDL-treated HUVECs	Regulation of FAS	Apoptosis, proliferation, and migration	[62]
MiR-663	↑	Ox-LDL-treated VSMCs and ApoE^−/−^ mice	Regulation of HMGA2	Inflammation and proliferation	[63]
MiR-320a	↑	Ox-LDL-treated VSMCs	Regulation of RGS5	Promoting migration and proliferation and reducing apoptosis	[64]
MiR-129-5p	↓	Ox-LDL-treated A7r5 cells	HMGB1/PI3K/AKT pathway	Reduction in migration	[65]
LncRNA TPRG1-AS1	↑	Human aortic smooth muscle cells and ApoE knockout mice	Regulation of MYH9	Migration and neointimal formation	[66]
LncRNA FGF7-5 and lncRNA GLRX3	↑	Carotid plaque of atherosclerotic patients and ox-LDL-treated HUVECs	MiR-2681-5p/ERCC4 pathway	Reduction in atherosclerosis-induced apoptosis	[67]
LncRNA HOXA11-AS	↓	Ox-LDL-treated HUVECs and ApoE knockout mice	MiR-515-5p/ROCK1 pathway	Proliferation, apoptosis, and dephosphorylation of eNOS	[68]
LncRNA H19	↑	Ox-LDL-treated human aortic endothelial cells (HAECs)	MiR-152/VEGFA pathway	Proliferation, migration, and tube formation	[69]
LncRNA DANCR	↑	Human serum and VSMCs	Regulation of miR-335-5p	Proliferative abilities and migration capacities	[70]
LncRNA RMST	↑	Human serum and ox-LDL-treated HUVECs	Regulation of miR-224-3p	Inflammation	[71]
LncRNA PVT1	↑	Human serum and ox-LDL-treated HUVECs	Regulation of miR-30c-5p	Proliferation, apoptosis, and inflammation	[72]
LncRNA XIST	↑	Ox-LDL-treated VSMCs	MiR-539-5p/SPP1 pathway	Proliferation and migration	[54]
LncRNA MDRL	↓	MAVSMCs and LDLR knockout mice with high-fat diet	MiR-361/SQSTM1/NLRP3 pathway	Attenuation of apoptosis and inflammation	[55]
Circ_0021155	↑	Ox-LDL-treated VSMCs	MiR-4459/TPRM7 pathway	Proliferation, migration, and phenotypic transformation	[57]
Circ_0086296	↑	Human carotid plaque, ox-LDL-treated HUVECs, and ApoE knockout mice	MiR-576-3p/IFIT1/STAT1 pathway	Proliferation, migration, and inflammation	[56]
Circ_0024103	↑	Ox-LDL-treated HUVECs	MiR-363/MMP-10 pathway	Migration, tube formation, and apoptosis	[73]
Circ_0002194	↑	Ox-LDL-treated HUVECs	MiR-637/PACS2 pathway	Apoptosis and oxidative stress	[74]
Circ_0005699	↑	Ox-LDL-treated HUVECs and ApoE knockout mice	MiR-450b-5p/NFKB1 pathway	Apoptosis and inflammation	[75]
Circ_PTPRA	↑	Human serum and ox-LDL-treated HUVECs	Regulation of miR-671-5p	Apoptosis and inflammation	[76]
Circ_NMD3	↓	Ox-LDL-treated HUVECs	MiR-498/BAMBI pathway	Attenuation of proliferation and apoptosis	[77]
Circ_0093887	↓	Ox-LDL-treated HAECs	MiR-758-3p/BAMBI pathway	Apoptosis and inflammation	[78]
Hsa_circ_0030042	↑	TNF-α-treated VSMCs	MiR-514a-3p/FOXO1 pathway	Proliferation, migration, and apoptosis	[79]
Hsa_circ_0008896	↑	Ox-LDL-treated VSMCs	MiR-633/CDC20B pathway	Proliferation and migration	[80]
Circ_CHFR	↑	Human serum and ox-LDL-treated HUVECs	MiR-15b-5p/GADD45G pathway	Apoptosis and inflammation	[81]
Circ_ARHGAP12	↑	Ox-LDL-treated MAVSMCs and ApoE knockout mice	MiR-630/EZH2/TIMP2 pathway	Regulation of AS progression	[82]

**Table 2:** Noncoding RNAs in cardiac hypertrophy (CH).

NcRNA	Expression in CH	Experimental Models	Mechanisms of Action	Roles	Reference
MiR-212	↑	PE- and Ang II-treated NRVCs and a rat model of abdominal aortic constriction	Regulation of TCF7L1 a	Cardiac hypertrophy	[109]
MiR-21	↑	Ang II-treated NRVCs and miR-21 knockout mice	S100a8/NF-κB/NFAT pathway	Cardiac hypertrophy	[110]
MiR-143-3p	↑	Female mice with obesity- induced cardiac hypertrophy	Sox6/Myh7 pathway	Obesity-induced cardiac hypertrophy	[111]
MiR-30d	↓	PE- and Ang II-treated hypertrophy in NRVCs and ISO-treated rats	MAP4K4/GRP78 a/NFAT pathway	Attenuation of pathological hypertrophic changes	[107]
MiR-27a-3p	↑	Human plasma and Ang II-treated H9C2 and mice	Regulation of NOVA1	Cardiac hypertrophy	[112]
MiR-204-5p	↑	Stretch-induced H9C2 and NRVCs, and TAC in miR-204 knockout mice	APJ signaling pathway	Attenuation of cardiac hypertrophy	[113]
MiR-410-3p	↑	Ang II-treated NRVCs	Regulation of Smad7	Cardiac hypertrophy	[114]
MiR-339-5p	↑	ISO-induced NRVCs	VCP/mTOR/S6K pathway	Cardiac hypertrophy	[115]
MiR-27b-3p	↑	MiR-27b knockout mice subjected to TAC and AngII	Regulation of FGF1	Cardiac hypertrophy	[116]
LncRNA MHRT	↓	Ang II-treated NRVCs and TAC-induced mice	SUMOylation of SIRT1 activating PGC1-α/PPAR-α pathway	Attenuation of cardiac hypertrophy	[117]
LncRNA MALAT1	↓	Ang II-treated NRVCs	MiR-181a/HMGB2 axis	Attenuation of cardiac hypertrophy	[118]
LncRNA NBR2	↓	Human plasma and Ang II- treated AC16	LKB1/AMPK/SIRT1 pathway	Attenuation of cardiac hypertrophy	[119]
LncRNA MIAT	↑	Ang II-treated NRVCs and TAC-induced mice	YTHDF2/PPARα/CPT-1a pathway	Cardiac hypertrophy	[120]
LncRNA TINCR	↓	Ang II-treated H9C2 and TAC-induced mice	MiR-211-3p/VEGFB/SDF-1α/CXCR4 pathway	Attenuation of cardiac hypertrophy	[105]
LncRNA H19	↓	Human serum and ISO-treated mice	MiR-145-3p/SMAD4 pathway	Attenuation of cardiac hypertrophy	[121]
LncRNA RMRP	↑	Human cardiac hypertrophic tissues and PE-treated cardiomyocytes	Regulation of miR-1	Cardiac hypertrophy	[122]
Circ_0001006	↑	Ang II-treated NRVCs and TAC-induced mice	MiR-214-3p/PAK6 axis	Cardiac hypertrophy	[108]

**Table 3:** Noncoding RNAs in myocardial infarction (MI).

NcRNA	Expression in MI	Experimental Models	Mechanisms of Action	Roles	Reference
CircTMEM165, circUBAC2, circZNF609, circANKRD12, and circSLC8A1	↑	Blood of human MI patients and H_2_O_2_-induced oxidative stress model in AC-16 cells	Several miRs	Apoptosis and diagnostic biomarker	[123]
LncRNA APF	↑	Blood of human MI patients	Targeting autophagy via miR-188-3p	Diagnostic biomarker	[131]
LncRNA BACE1-AS	↑	Blood mononuclear cells derived from CAD patients	Undetermined	Diagnostic biomarker	[132]
LncRNA CAIF	↓	Mouse MI model and H_2_O_2_- induced oxidative stress model in mouse cardiomyocytes	CAIF/miR-488-5p/AVEN axis	Apoptosis	[133]
LncRNA EPS	↓	Mouse MI model and HL-1 cells treated in oxygen and glucose deprivation (OGD)	Maintaining MYH6 stability through recruitment of HNRNPL	Inflammation and apoptosis	[134]
LncRNA LINC00461	↑	Mouse I/R injury model	MiR-185-3p/Myd88 axis	Apoptosis	[135]
LncRNA MALAT1	↑	Blood of human MI patients	Undetermined	Prognostic biomarker	[136]
LncRNA MALAT1	↑	Monocytes of human MI patients	Undetermined	Diagnostic biomarker	[126]
LncRNA MALAT1	↑	Plasma of human STEMI patients	Sponging miR-30e, miR-126, and miR-155	Biomarker to diagnose no flow	[124]
LncRNA MALAT1	↑	Mouse I/R injury model and H9c2 and HL-1 cells subjected to hypoxia/reoxygenation	PI3K/AKT/eNOS signaling via miR-133a-3p	Apoptosis	[127]
LncRNA MBNL1-AS1	↑	Rat MI models and H9c2 cells treated by hypoxia	MiR-132-3p/SOX4 axis	Apoptosis	[137]
LncRNA MCM3AP-AS1	↑	Rat MI models and knockdown in vascular ECs (VECs)	MiR-24-3p/EIF4G2 pathway	Promoting proliferation and migration of VECs	[138]
LncRNA MIAT	↑	H9C2 cells subjected to hypoxia	SF1/CGRP pathway	Pyroptosis	[139]
LncRNA MIR4435-2HG	↑	Human MI patients, mouse I/R model, and H_2_O_2_-induced oxidative stress model	MiR-125a-5p/MTFP1 pathway	Apoptosis	[140]
LncRNA HOTAIR	↑	Mouse MI model and cardiomyocytes subjected to hypoxia/reoxygenation	MiR-206/FN1 axis	Apoptosis	[141]
LncRNA PVT1	↑	Plasma of patients with good coronary collateral circulation, HUVECs, and mouse hind limb ischemia and MI models	PVT1/miR-15b-5p/AKT3 axis	Angiogenesis	[142]
LncRNA SNHG1	↓	AC-16 cells subjected to hypoxia/reoxygenation	MiR-450b-5p/IGF1 axis	Apoptosis	[143]
LncRNA TTTY15 and LncRNA HULC	↑	Plasma of human MI patients	Undetermined	Biomarkers to diagnose AMI	[144]
MiR-150	↓	Mouse MI model and HCFs	MIAT/miR-150/HOXA4 pathway	Blunting CF activation	[129]
MiR-223 and miR-186	↑	Blood of human MI patients	Undetermined	Prognostic biomarkers	[125]
MiR-411	-	Mouse MI model and NRVCs	Hippo/YAP pathway	Cardiomyocyte proliferation and survival	[128]

**Table 4:** Noncoding RNAs in pulmonary hypertension (PH).

NcRNA	Expression in PH	Experimental Models	Mechanisms of Action	Roles	Reference
MiR-335	↑	DHA-treated hypoxic PH (HPH) mice and PASMCs	Regulation of Vangl2	PASMC proliferation in PH	[147]
MiR-503	↓	DHA-treated HPH mice and PASMCs	ELAVL2/miR-503/PI3K/AKT pathway	Reduced PASMC proliferation in PH	[148]
MiR-486-5p	↓	Hypoxia-induced human primary PASMCs	Smad2/3 pathway	hPASMC proliferation and migration in PAH	[154]
MiR-130	↓	PASMCs and FGF21-treated mice	FGF21/PPARγ axis	Inhibited PASMC proliferation and migration in PAH	[155]
MiR-126	↓	Primary HLMVECs	Regulation of ADAM9	Angiogenesis and pulmonary vascular remodeling in COPD-PH	[156]
MiR-27b-3p	↑	Monocrotaline (MCT)- induced PAH rats and rat PASMCs	FBXW7/KLF5/GLI1 pathway	PASMC proliferation and migration in PAH	[149]
MiR-21-5p	↑	MCT-induced PAH rats	Regulation of FilGAP	PASMC proliferation in PH	[157]
MiR-214-3p, miR-326-3p, and miR-125b-2-3p	↓	IUGR-induced PH rats	Regulation of FoxM1	PASMC proliferation and migration in PH	[158]
LncRNA H19	↓	FGF21-treated HPH mice	MTORC1/EIF4EBP1 pathway	Reduced PASMC proliferation in PH	[152]
LncPTSR	↓	Rat PASMCs and HPAH rats	Regulation of PMCA4 and intracellular Ca^2+^	Reduced PASMC proliferation in PAH	[159]
LncRNA SOX2-OT	↓	Hypoxia-induced human PASMCs (hPASMCs)	MiR-455-3p/SUMO1 pathway	Attenuating hPASMC proliferation, migration, antiapoptosis, and inflammation in PAH	[153]
CircGSAP	↓	MCT-induced PH rats and CO_2_-treated human PMECs	MiR-27a-3p/BMPR2 pathway	Reduced PMEC proliferation, migration, and increased cell mortality in IPAH	[150]
CircSIRT1	↑	MCT-induced PH rats and PASMCs	MiR-145-5p/AKT3 axis	PASMC proliferation and migration in PH	[160]

## Data Availability

Not applicable.
